# Systemic Anticancer Therapy Timelines Extraction From Electronic Medical Records Text: Algorithm Development and Validation

**DOI:** 10.2196/67801

**Published:** 2025-09-03

**Authors:** Jiarui Yao, Eli Goldner, Harry Hochheiser, Sean Finan, John Levander, David Harris, Piet C de Groen, Elizabeth Buchbinder, Danielle Bitterman, Jeremy L Warner, Guergana Savova

**Affiliations:** 1 Computational Health Informatics Program, Boston Children’s Hospital, Harvard Medical School, 401 Park Drive, Boston, MA, 02115, United States, 1 7813545014; 2 Department of Biomedical Informatics, University of Pittsburgh, Pittsburgh, PA, United States; 3 Department of Medicine, Division of Gastroenterology, Hepatology and Nutrition, University of Minnesota, Minneapolis, MN, United States; 4 Department of Medical Oncology, Dana Farber Cancer Institute, Boston, MA, United States; 5 Artificial Intelligence in Medicine (AIM) Program, Mass General Brigham, Harvard Medical School, Boston, MA, United States; 6 Department of Radiation Oncology, Brigham and Women’s Hospital/Dana-Farber Cancer Institute, Boston, MA, United States; 7 Center for Clinical Cancer Informatics and Data Science, Legorreta Cancer Center, Brown University, Providence, RI, United States; 8 Brown University Health Cancer Institute, Rhode Island Hospital, Providence, RI, United States

**Keywords:** systemic anticancer therapy, electronic medical records, treatment timelines extraction, natural language processing, large language models

## Abstract

**Background:**

The systemic treatment of cancer typically requires the use of multiple anticancer agents in combination or sequentially. Clinical narrative texts often contain extensive descriptions of the temporal sequencing of systemic anticancer therapy (SACT), setting up an important task that may be amenable to automated extraction of SACT timelines.

**Objective:**

We aimed to explore automatic methods for extracting patient-level SACT timelines from clinical narratives in the electronic medical records (EMRs).

**Methods:**

We used two datasets from two institutions: (1) a colorectal cancer (CRC) dataset including the entire EMR of the 199 patients in the THYME (Temporal Histories of Your Medical Event) dataset and (2) the 2024 ChemoTimelines shared task dataset including 149 patients with ovarian cancer, breast cancer, and melanoma. We explored finetuning smaller language models trained to attend to events and time expressions, and few-shot prompting of large language models (LLMs). Evaluation used the 2024 ChemoTimelines shared task configuration—Subtask1 involving the construction of SACT timelines from manually annotated SACT event and time expression mentions provided as input in addition to the patient’s notes and Subtask2 requiring extraction of SACT timelines directly from the patient’s notes.

**Results:**

Our task-specific finetuned EntityBERT model achieved 93% *F*_1_-score, outperforming the best results in Subtask1 of the 2024 ChemoTimelines shared task (90%). It ranked second in Subtask2. LLM (LLaMA2, LLaMA3.1, and Mixtral) performance lagged the task-specific finetuned model performance for both the THYME and shared task datasets. On the shared task datasets, the best LLM performance was 77% macro *F*_1_-score, 16% points lower than the task-specific finetuned system (Subtask1).

**Conclusions:**

In this paper, we explored approaches for patient-level timeline extraction through the SACT timeline extraction task. Our results and analysis add to the knowledge of extracting treatment timelines from EMR clinical narratives using language modeling methods.

## Introduction

The systemic treatment of cancer typically requires the use of multiple anticancer agents in combination or sequentially. Systemic anticancer therapy (SACT), which includes traditional cytotoxic chemotherapy, endocrine therapy, targeted therapy, and immunotherapy, has both a low therapeutic index as well as synergistic potential when agents are given in combination. Due to cumulative toxicities, the order in which SACT components are received is much more important than only whether individual drug exposures happened or not, whether in the curative or noncurative setting. Furthermore, patients may receive an extended sequence of treatments across multiple health care settings, systems, and insurance arrangements, making an accurate tally of the totality of treatment using standard structured data resources extremely challenging if not impossible. Meanwhile, clinical narrative texts often contain extensive descriptions of the temporal sequencing of SACT, setting up an important task that may be amenable to automated extraction approaches.

Clinical natural language processing (NLP) is a field that builds computational methods to enable machines to process clinical narratives. Temporality has been a key research area within clinical NLP as it has a wide range of applications including temporal sequencing of SACT [1]. The focus of temporality extraction in clinical NLP has been mainly on instance-level pairwise temporal relation extraction from electronic medical records (EMRs). Instance-level pairwise temporal relations (TLINKs) are the links between an event (EVENT) mention and a temporal expression (TIMEX3) mention or between two event mentions, constituting a triple of the TLINK and the other two components. The set of TLINKs values, that is, type of temporal relations, is CONTAINS, BEFORE, OVERLAP, BEGINS-ON, ENDS-ON, and NOTED-ON [1]. The event that CONTAINS another event is referred to as a narrative container (CONTAINS-1 is the reverse of CONTAINS, meaning an EVENT is contained by the narrative container). In addition, each EVENT has a temporal relation with the document creation time (DocTimeRel), one of BEFORE, BEFORE-OVERLAP, OVERLAP, or AFTER.

The construction of benchmarks, such as THYME (Temporal Histories of Your Medical Event) and i2b2 [1,2], along with the SemEval shared tasks [3-6] on temporality advanced the methodologies and established the state-of-the-art (SOTA) for the task [7-12]. The sophisticated SOTA methods for temporal relation extraction open the door for exploring automatic patient-level timeline construction.

**Figure 1.**
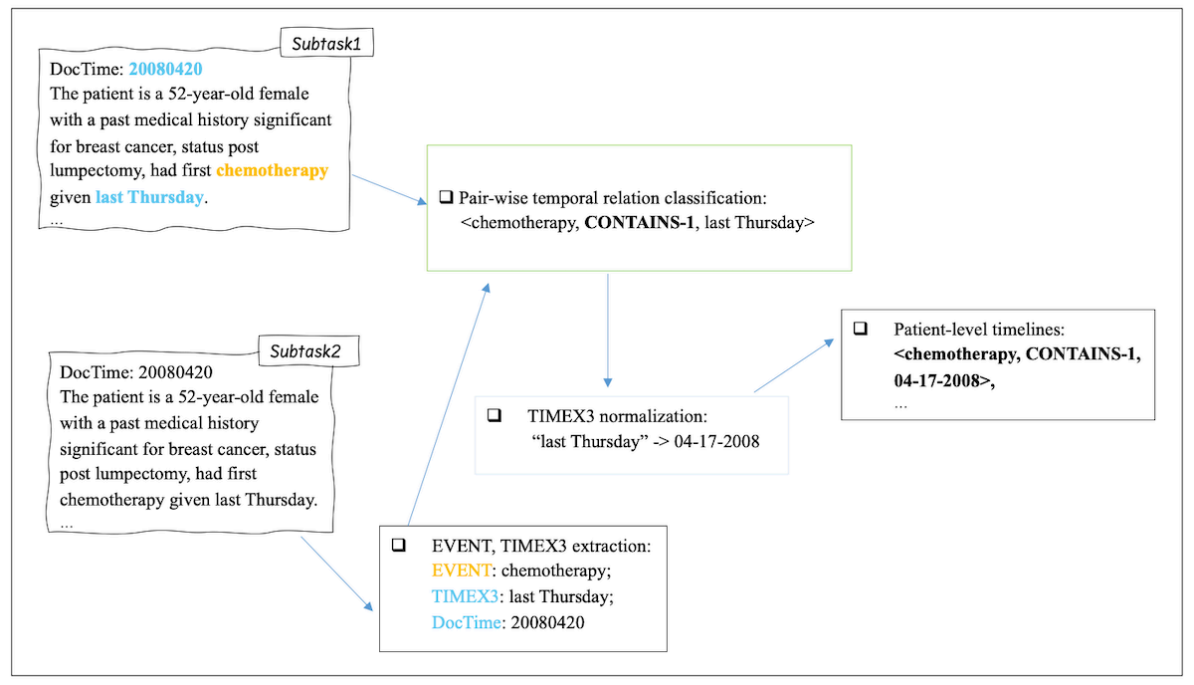
Summary of the 2024 ChemoTimelines shared task. TIMEX3: time expressions; CONTAINS-1: reverse of CONTAINS, meaning “chemotherapy” is contained by “last Thursday”; DocTime: document creation time.

The 2024 ChemoTimelines shared task [13] formulated SACT timeline construction as an information extraction task and provided the deidentified free text documents (except for dates) from the EMRs of 57,520 (breast and ovarian cancer) and 15,946 (melanoma) patients from University of Pittsburgh Medical Center. The documents represented a wide variety of notes, for example, pathology reports, clinical notes, radiology reports, emergency department visits, discharge summaries, etc. A subset of 149 patients was expert-annotated for EVENT mentions, TIMEX3 mentions, and instance-level pairwise temporal relations following the THYME2 schema [1,14] and patient-level timelines of SACT events. The shared task offered 2 subtasks. “Subtask1” involved creating timelines from gold EVENTS and TIMEX3 mentions. “Subtask2” challenged the participants to build end-to-end systems that extracted patient-level SACT timelines directly from the free texts. In this work, “end-to-end” means all text processing is done automatically. Figure 1 summarizes the 2 subtasks. Various approaches were explored by the shared task participants—from supervised finetuning [15,16] to LLM prompting [17,18]. The impressive results (*F*_1_-score=90 for Subtask1 and *F*_1_-score=70 for Subtask2) achieved by the systems from top participants [15] demonstrated the usability and effectiveness of NLP models for this task. The top systems implemented task-specific finetuning of smaller pretrained language models (LMs). Specifically, the LAILab system [15] cast the task as a sequence-to-sequence task, and finetuned Flan-T5-XXL [19] and BART-large [20]. It achieved the best results in the shared task for both subtasks. The Wonder system [16] generated synthetic data using GPT-4 for data augmentation, then finetuned BioLM [21]. The baseline system offered by the organizers [13] also took the supervised finetuning approach with PubMedBERT [22] and secured the second place in both subtasks. In the rest of the paper, for simplicity, we refer to the 2024 ChemoTimelines shared task as the shared task.

In this paper, we further researched SACT timeline extraction using the shared task dataset and adding the dataset of another frequent type of cancer (such as CRC) from another academic medical center. We explored task-specific finetuning approaches and LLM prompting [23-29] to extract SACT timelines. We compared our results on the breast, ovarian, and melanoma datasets from the shared task to the results of the shared task participants. We achieved a new SOTA in Subtask1. We established the SOTA for the CRC dataset as this is a new dataset. Our LLM-based system investigations add to the research of using LLMs for end-to-end SACT treatment timeline extraction from clinical narratives, as only one team explored end-to-end timeline extraction using LLMs in the shared task.

The contributions of this paper are as follows.

First, the approaches for patient-level timeline extraction through the task of SACT timeline extraction. We perform experiments on the 2024 ChemoTimelines shared task as well as on the THYME CRC patients. Our results and analysis on this task add to the knowledge of extracting treatment timelines from EMRs using LLM-based methods.

Second, the SOTA performance of our finetuned LM-based system for Subtask1 of the 2024 ChemoTimelines shared task.

Third, SOTA performance with LLM prompting approaches for Subtask1 and Subtask2 of the 2024 ChemoTimelines shared task outperformed the shared task participant systems that took the approach of prompting LLMs.

## Methods

### Ethical Considerations

All electronic health record (EHR) data used in this study are deidentified in accordance with the datasets’ relevant privacy regulations [1,13,14]. We strictly adhered to the terms outlined in the data use agreement, ensuring that no data were transmitted to any external or public APIs. Ethics approval was not required because the study used secondary data that was aggregated and anonymized before analysis. All experiments were conducted on a secure local machine operating behind a firewall, maintaining full data confidentiality and integrity throughout the study.

### Tasks and Datasets

The first dataset we used was from the shared task [13]. The EMR notes of 149 patients with breast, ovarian, and melanoma cancers from the University of Pittsburgh Medical Center were expert-annotated by the shared task organizers for instance-level pairwise temporal relations following the THYME2 schema [1,14] and SACT patient-level timelines.

The second dataset we used included the THYME patients—199 CRC patients from Mayo Clinic. This dataset was NOT part of the 2024 ChemoTimelines shared task. Note that the original THYME corpus consisted of one radiology, one pathology, and one oncology note for each of the 199 CRC patients—not sufficient to extract SACT timelines. Therefore, for the work described in this paper, we obtained the entire EMR documentation for these 199 CRC patients (all manually deidentified except for dates). As with the shared task patients, the CRC patient EMRs were represented by a wide variety of document types. Following the shared task protocol, the CRC notes were expert-annotated for instance-level pairwise temporal relations following the THYME2 schema and SACT patient-level timelines. Table 1 shows the dataset distributions. Table S1 in Multimedia Appendix 1 provides the pairwise label distributions. The label set for the pairwise relations is CONTAINS, BEGINS-ON, ENDS-ON, OVERLAP, and BEFORE. In the final SACT timeline, we converted CONTAINS to CONTAINS-1 so that all triples are structured as <EVENT, TLINK, TIMEX3>, where CONTAINS-1 semantically indicates that the drug was administered on the date specified by the temporal expression (TIMEX3). Note that we did not use i2b2 2012 because we focused on cancer treatment timeline extraction only in this work. Textbox 1 presents a concrete example of patient-level SACT timelines.

As is the established convention, in this paper, we refer to the labels in the shared task and THYME datasets as “gold.” All datasets come with predefined training (train), development (dev), and test splits that we used accordingly. Note that the gold labels of the shared task test set were not publicly available; however, participants could submit their system predictions to the shared task organizers to get evaluation results, thus providing independent evaluation over a held-out, eyes-off dataset.

**Table 1.** Dataset summary.

Splits	Patients	Notes	Words^a^	EVENT mentions	TIMEX3^b^ mentions	TLINKs^c^
Ovarian cancer (from 2024 ChemoTimelines shared task)						
Train	26	1675	1,183,632	1168	597	494
Dev^d^	8	562	308,814	790	312	226
Test	8	559	257,116	664	381	Not released^e^
Breast cancer (from 2024 ChemoTimelines shared task)						
Train	33	1002	465,644	1023	576	455
Dev	16	499	225,588	279	146	113
Test	35	1333	786,896	2560	1118	Not released
Melanoma (from 2024 ChemoTimelines shared task)						
Train	10	233	124,924	147	78	48
Dev	3	211	178,308	789	261	201
Test	10	229	156,083	398	193	Not released
Colorectal cancer (CRC)						
Train	98	12,990	6,038,431	11,161	6155	5897
Dev	50	6810	3,105,675	3964	2194	1924
Test	51	7357	3,587,387	7552	3612	4403

a “Words” denotes tokens delimited by white spaces.

b TIMEX3: time expressions.

c TLINKs: pairwise temporal relations.

d Dev: development set.

e Note that the number of test set TLINKs for the 2024 ChemoTimelines shared task was not released publicly.

Textbox 1. **An example of a summarized patient-level SACT timeline extracted from the entire patient’s EMR chart.**

- ['chemotherapy', 'contains-1', '2013-06-20']
- ['carboplatin', 'contains-1', '2013-10-24']
- ['carboplatin', 'contains-1', '2013-09-19']
- ['carboplatin', 'contains-1', '2013-07-18']
- ['carboplatin', 'contains-1', '2013-08-08']
- ['carboplatin', 'contains-1', '2013-08-29']
- ['taxol', 'contains-1', '2013-10-24']
- ['taxol', 'contains-1', '2013-09-19']
- ['taxol', 'contains-1', '2013-07-18']
- ['taxol', 'contains-1', '2013-08-08']
- ['taxol', 'contains-1', '2013-08-29']

### Approaches

**Figure 2.**
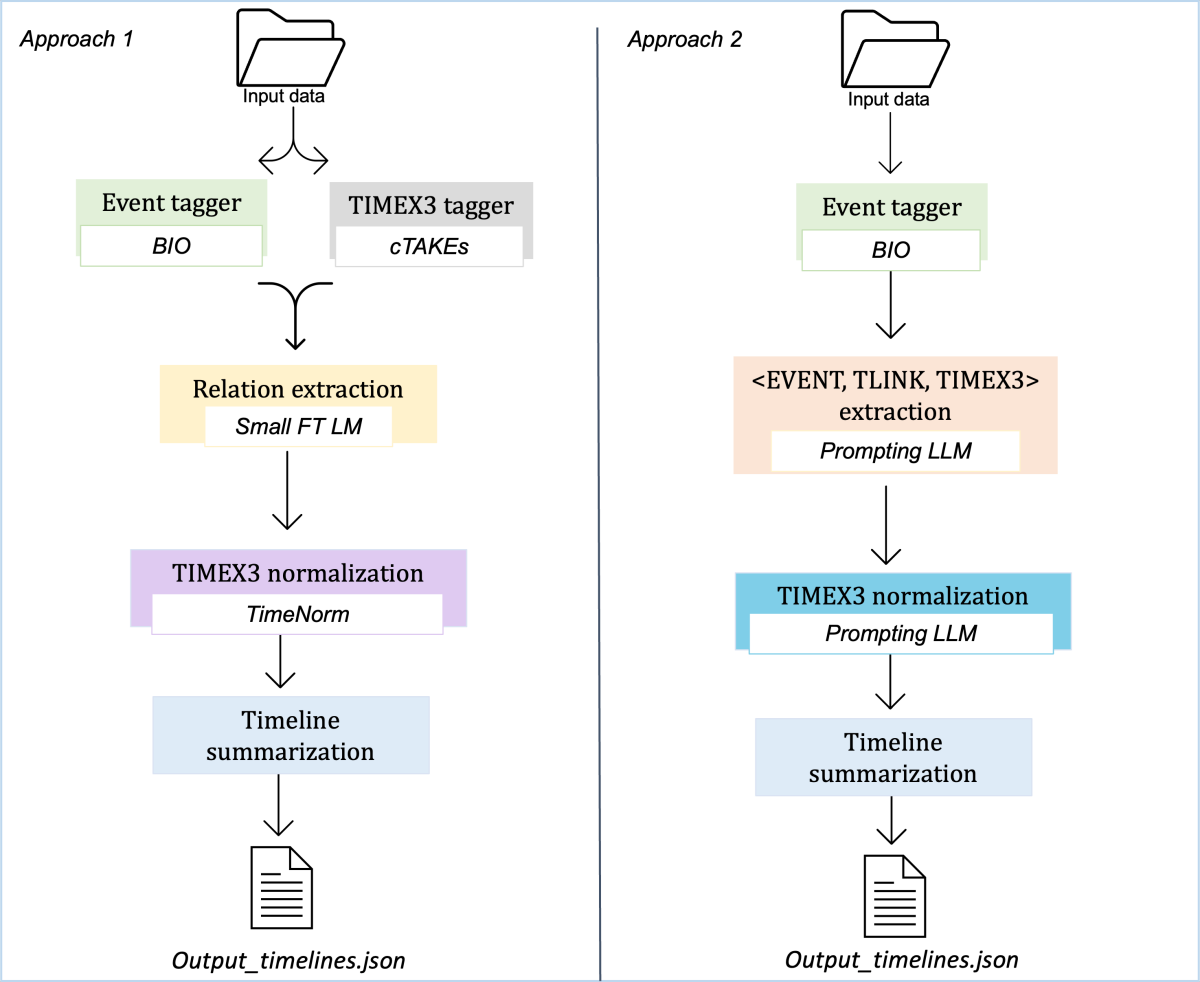
Methods summary. On the left-hand side, temporal relations are classified via a small finetuned language model (FT LM). On the right-hand side, temporal relation triplets are extracted by prompting large language models (LLMs). In both approaches, EVENTS are extracted using a **B**egin-**I**nside-**O**utside (BIO) tagger. Output for both systems is the same, see Textbox 1. cTAKES: Apache Clinical Text Analysis and Knowledge Extraction System; TIMEX3: time expressions; TLINK: pairwise temporal relation.

We explored 2 approaches for the task of SACT timelines extraction: (1) finetuning smaller LMs and (2) prompting LLMs. Figure 2 shows the complete pipeline of both approaches. We describe each approach in detail in this section.

#### Approach 1: Finetuning LMs for Temporal Relation Extraction

##### Overview

In this approach, we cast the task of SACT timeline extraction as a pairwise temporal relation extraction task followed by a temporal relation summarization step. Given input texts, we designed a pipeline with the following steps: (1) extracting SACT EVENT mentions, (2) extracting TIMEX3 mentions, (3) classifying pairwise EVENT-TIMEX3 temporal relations, (4) normalizing TIMEX3 mentions, and (5) summarizing and refining patient-level timelines.

##### Extracting SACT EVENT Mentions

We trained a sequence labeling tagger that marks the beginning, inside, and outside (BIO) of a SACT treatment EVENT mention in the text. The tagger was trained on the train split of the gold labeled data by finetuning a pretrained LM [22,30]. The “Experimental Settings” section shows more details.

##### Extracting TIMEX3 Mentions

TIMEX3 mentions were extracted by the temporal module of the Apache Clinical Text Analysis and Knowledge Extraction System (cTAKES) [31], a publicly available text processing system. The precision, recall, and *F*_1_-scores of cTAKES for TIMEX3 mention extraction are 57.17%, 83.95%, and 67.25%, respectively; evaluated on the original THYME dataset described in the “Tasks and Datasets” subsection. Different methodologies were used for SACT EVENT mention extraction and TIMEX3 mention extraction because there was no publicly available SACT EVENT extractor with solid performance at the time of the experiments.

##### Classifying Pairwise EVENT-TIMEX3 Temporal Relations

Given an EVENT-TIMEX3 pair, the task is to determine the temporal relation between them according to a predefined label set of TLINKs (described in the “Introduction” and “Tasks and Datasets” sections). For example, if the patient started a regimen of Taxol on August 1, 2012, the relation between “Taxol” and “August 1, 2012” is BEGINS-ON. Inspired by previous works [11], we finetuned EntityBERT for this step to create an LM specifically trained to attend to EVENT and TIMEX3 mentions. The input to the model was the EVENT and TIMEX3 mentions within a context window with the EVENT and TIMEX3 mentions highlighted by special tokens, possibly crossing sentence boundaries. We followed the same data preprocessing format as described in [7,9,11]. Concretely, EVENT and TIMEX mentions are highlighted by XML tags “<e>,” “</e>,” “<t>,” and “</t>.” The context window that defines the token distances between an EVENT and TIMEX3 in an EVENT-TIMEX3 pair is set to 60 tokens, empirically derived to cover over 95% of the EVENT-TIMEX3 pair instances. The model was trained on the train split of the gold-labeled data for multiclass classification.

##### Normalizing TIMEX3 Mentions

The goal of this step is to map TIMEX3 mentions to a computable format. We used TimeNorm [32,33] to normalize the TIMEX3 mentions and the document creation time (DocTime) to ISO-TimeML standard [34] (eg, “yesterday” in a note with a DocTime of “2022-04-29” would be normalized to “2022-04-28”).

##### Summarizing and Refining Patient-Level Timelines

A patient’s SACT history can be mentioned in multiple notes in different contexts. For example, the physician may discuss the termination of one treatment due to side effects; despite that, in another note, they may say that the therapy will be given to the patient for 3 more cycles. Therefore, after the instance-level temporal relation extraction, deduplication and conflict resolution are necessary to get the final patient-level SACT timelines. For this step, we followed the heuristics from the shared task [13].

### Approach 2: Prompting LLMs for SACT Timeline Extraction

#### Overview

We developed an end-to-end timeline extraction pipeline via LLM prompting. This pipeline involved two steps: Step 1 focused on extracting <EVENT, TLINK, TIMEX3> triplets from clinical texts, and Step 2 was designed for TIMEX3 normalization. We took the approach of in-context learning, which refers to the method of adding exemplars of gold examples with answers to the prompt [25], a common practice in prompt engineering. Textbox 2 provides the prompt templates we used in both steps. For Step 1, we provide 4 exemplars for each TLINK label. For Step 2, we provide 5 exemplars in total. The exemplars are selected from the training split of the data. We explored the discrete prompting strategy where the prompts are created manually, ultimately settling on the prompts with the best performance.

Textbox 2. **Prompt templates used in our large language model (LLM) experiments. For Step 1, we provide 4 exemplars for each label. For Step 2, we provide 5 exemplars in total.**

- Step 1 prompt: You are a helpful assistant for oncologists. You will read the given PATIENT EHR and summarize the patient's chemotherapy treatment TIMELINES. Please only output TIMELINES in the requested format. Please do not include any other text or reasoning, do not include timelines for any other treatments besides chemotherapy. Please do not use any labels other than the ones given in the examples, i.e., BEGINS-ON, ENDS-ON, CONTAINS. Here are some examples.
- Step 2 prompt: You are asked to decide the date of a time expression. If today was 2013-05-02, what would the date of yesterday be? Please only output the date in the format of “YYYY-MM-DD”. Answer “Unknown” if you don't know. Here are some examples.

#### Step 1: Extracting < EVENT, TLINK, TIMEX3> Triplets

The construction of patient-level treatment timelines requires the system to process all notes of a patient, thus the input can exceed the LLM context window. Current open LLMs have a limited number of tokens they can process per time, for example, LLaMA1 [35] supports up to 2048 tokens and LLaMA2 [23] supports up to 4096 tokens; however, even if the LLM could ingest all the notes of one patient as input per time, which would not be an efficient way of processing texts as transformers’ self-attention scales quadratically with input length. Therefore, sending all the notes of a patient to LLMs at one time is not practical. To make this task more feasible for LLMs, we prompted the LLM with only relevant snippets of notes and assembled the timelines afterwards. Specifically, we extracted SACT EVENT mentions using the BIO tagger trained in Approach 1, then fed the LLM the sentences containing the SACT EVENT mentions to extract the triplets. Note, the input to the LLM was a sentence, unlike the context window instances fed to the pairwise classifier in Approach 1. In our initial experiments, we used context window instances with the LLMs as well; however, the partial sentences confused them as tokens outside of the window are discarded. To give LLMs a self-contained input with a reasonable sequence length, we decided to give a complete sentence as input for LLMs instead of a context window as we did in Approach 1.

#### Step 2: TIMEX3 Normalization With LLMs

We applied in-context learning to normalize the TIMEX3 mentions. For each output triplet from Step 1, we prompted the model to normalize the date of the TIMEX3 mention given the DocTime of the note. We then assembled the final timelines, using the same heuristics as in Approach 1.

### Experimental Settings

We explored two approaches for the task of SACT timelines extraction: (1) finetuning smaller LMs and (2) prompting LLMs. For the first approach, we finetuned PubMedBERT base model [22] to train the SACT event tagger. For the temporal relation classification task, we finetuned EntityBERT based on the results reported by Lin et al [11], where they finetuned BioBERT, PubMedBERT, and EntityBERT for clinical temporal relation classification and found that EntityBERT outperformed the other two models. For the experiments with LLMs, we chose LLaMA2-70B [23], LLaMA3.1-70B [36], and Mixtral-8×7B-Instruct-v1 [24], which are current SOTA open LLMs. We did not use proprietary LLMs such as GPT4 [26] because we did not have access to their Health Insurance Portability and Accountability Act (HIPAA)-compliant versions. The open models we experimented with are reported to have yielded results competitive with those of the proprietary models [24]. Furthermore, we compare our results with those systems in the shared task for the types of cancers included in the shared task. For the CRC dataset (not included in the shared task), we establish the first result that will serve as the baseline for the community. See Table S2 in Multimedia Appendix 1 for details on the computational settings.

We experimented with prompting LLMs for both Subtask1 and Subtask2. In Subtask1, we provided explicit gold SACT events and time expressions in the text, then prompted the LLM to predict the temporal relation between them. The prompt template for this subtask is shown in Table S3 in Multimedia Appendix 1. In Subtask2, we passed to the LLM only plain text as input, then asked the LLM to extract the SACT events, time expressions, and temporal relation between them in 1 step. Textbox 2 lists the prompt template for Subtask2.

### Evaluation and Baseline

We used the evaluation metric provided by the shared task, which computed the average *F*_1_-scores across all patients. There were 4 settings with different temporal granularities: strict, relaxed-to-day, relaxed-to-month, and relaxed-to-year. For example, the relaxed-to-month setting required the model to correctly predict the year and month when the therapy was given, while the strict setting required the model to capture the exact date when the patient received the therapy. The official metric for the 2024 shared task was relaxed-to-month scores, which we used as our metric to report the main results in this paper. Results using other metrics are given in Table S4 in Multimedia Appendix 1.

As a baseline, we used the baseline system used in the shared task, which implemented a predefined dictionary as a lookup table for SACT EVENT extraction and a finetuned LM for temporal relation classification. We also compared our results on the 3 types of cancer (breast cancer, ovarian cancer, and melanoma) to the shared task leaderboard results.

## Results

In Table 2, we present our results on the development (Dev) and test sets. As the CRC dataset was not available for the shared task, we also present the results of our model finetuned only on the shared task data (under EntityBERT 3 Cr) for a direct comparison with other participating systems. That is, using Approach 1 described above, we trained 2 versions of the model. “EntityBERT” was trained on the shared task data and CRC data. “EntityBERT 3 Cr” was trained only on the shared task data (we combined the training datasets of multiple cancer types into 1 training dataset to train the EntityBERT 3 Cr model and EntityBERT model). Subtask1 in Table 2 shows the results with gold SACT EVENT and TIMEX3 mentions as input. In general, the finetuned EntityBERT and EntityBERT (3 Cr) outperformed LLaMA2, LLaMA3.1, and Mixtral LLMs by a large margin. Among the LLMs, LLaMA achieved higher scores than Mixtral. In Table 2, Subtask2 shows the end-to-end evaluation results. The SACT event extraction evaluation results using the BIO tagger can be found in Table S5 in Multimedia Appendix 1. We note a wide gap between the performance with gold mention input (Subtask1) and the performance with automatically extracted mentions (Subtask2), suggesting that the errors in the mention extraction stage propagate to the relation extraction stage and dramatically affect the overall accuracy of the system. We also notice that the smaller finetuned models outperform LLMs in most cases except for melanoma, the reasons for which we discuss in the Discussion section.

**Table 2.** Evaluation results of our systems across 4 types of cancers from 2 academic centers. Scores are macro *F*_1_-score, relaxed-to-month.

Cancer type and models		Subtask1^a^, %		Subtask2^b^, %	
		Development set	Test set	Development set	Test set
Ovarian cancer					
	EntityBERT^c^	93^e^	95^e^	64	61
	EntityBERT (3 Cr)^c^,^d^	93^e^	94	67^e^	69^e^
	LLaMA2^f^	70	70	29	42
	LLaMA3.1^g^	75	74	31	56
	Mixtral^h^	60	67	7	27
Breast cancer					
	EntityBERT^c^	97^e^	97	88^e^	63
	EntityBERT (3 Cr)^c^	97^e^	98^e^	87	66^e^
	LLaMA2	81	83	61	50
	LLaMA3.1	79	70	66	48
	Mixtral	66	63	37	25
Melanoma					
	EntityBERT^c^	86^e^	91^e^	43	39
	EntityBERT (3 Cr)^c^	86^e^	88	46	40
	LLaMA2	80	79	47^e^	47^e^
	LLaMA3.1	67	71	26	38
	Mixtral	65	65	4	25
Colorectal cancer (CRC)					
	EntityBERT^c^	90^e^	83^e^	58^e^	56^e^
	LLaMA2	66	77	40	32
	LLaMA3.1	66	68	45	38
	Mixtral	58	66	19	15

a Subtask1: input is gold entities (systemic anticancer therapy [SACT] events and time expressions).

b Subtask2: entities are automatically generated by the system.

c These are systems using small finetuned models.

d EntityBERT (3 Cr): EntityBERT model trained only on the shared task data.

e These are the best results.

f LLaMA2-70B.

g LLaMA3.1-70B.

h Mixtral-8×7B-Instruct-v1.

Furthermore, unlike the LLM prompting approaches, both our systems based on the smaller finetuned models can be deployed for inference on a laptop without a GPU. Our Subtask1 system is able to process approximately 14 notes/minute. Our Subtask2 system is able to process approximately 10 notes/minute. Assuming a typical patient with 200 notes, our Subtask1 system takes on average 14.5 minutes to process all of the patient’s notes, and our Subtask2 system takes on average 20 minutes to process all of the patient’s notes. On the other hand, the LLM prompting experiments were conducted on NVIDIA A100 GPUs. It took the LLaMA3.1 70B model approximately 28 minutes for Subtask1 and 13 minutes for Subtask2 to process 200 notes. It took LLMs less time to complete Subtask2 because only sentences containing TIMEX3 mentions needed to be processed in Subtask2.

**Figure 3.**
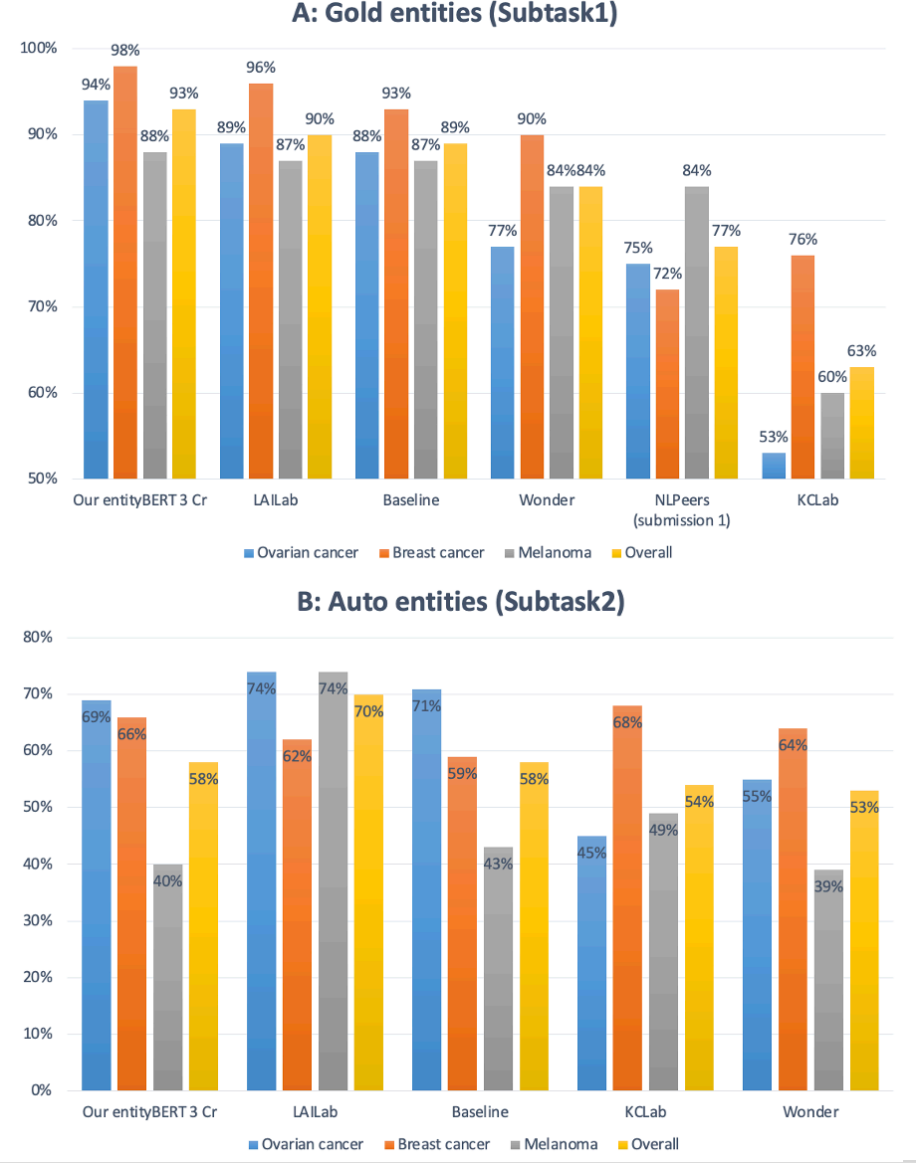
Comparison to finetuning-based models in the shared task [13,15,16,18,37]. Scores are relaxed-to-month macro *F*_1_-score on the test set. “Our EntityBERT, 3 cr” refers to the EntityBERT model trained only on the shared task data. The best-performing team in the shared task was LAILab [15].

We position our systems within the broader context of the 2024 ChemoTimelines shared task by comparing them with the shared task participants’ systems. If 1 shared task participant has multiple submissions, we take their best result for comparison. Note the official metric for the leader board is relaxed-to-month scores on the Test set. We first compare the result of our EntityBERT (3 Cr) model with the results of the participating systems using similar approaches, that is, finetuning smaller LMs [13,15,16,18,37]. In Figure 3-Part A, we can see that in Subtask1 our model achieved the best results overall and on the individual cancer types. Our Subtask1 result was 3 points higher than the best shared task score achieved by LAILab [15] (93% vs 90%). In Subtask2 (Figure 3-Part B), our system had the second-best overall scores. However, it is worth noting that LAILab finetuned Flan-T5-XXL [19], a model with 11B parameters, which was much bigger than the EntityBERT model we used that had about 100 million parameters.

Finally, we observe in Table 2 that the model trained only on the breast, ovarian, and melanoma data from the train split of the shared task (ie, EntityBERT 3 Cr) performed slightly better than its counterpart trained on the full train split containing all 4 types of cancer (ie, EntityBERT) in Subtask2. We conjecture that since there was more data for CRC than the other types of cancer within our dataset, the representation of the signal from the CRC data overwhelmed that of the other three cancer types inside the model. The addition of the second dataset (CRC) in this work aims to create a larger pool of datapoints adding a new type of cancer and a different institution as the data source. It also helps answer the questions of whether (1) a model built off data across different EMR sources might be feasible and (2) the quantity of the data matters. Our experiments on these two datasets show that (1) it is likely that institution-specific models capture treatment patterns better but not by a large margin and (2) patterns of the data-rich source likely dominate.

**Figure 4.**
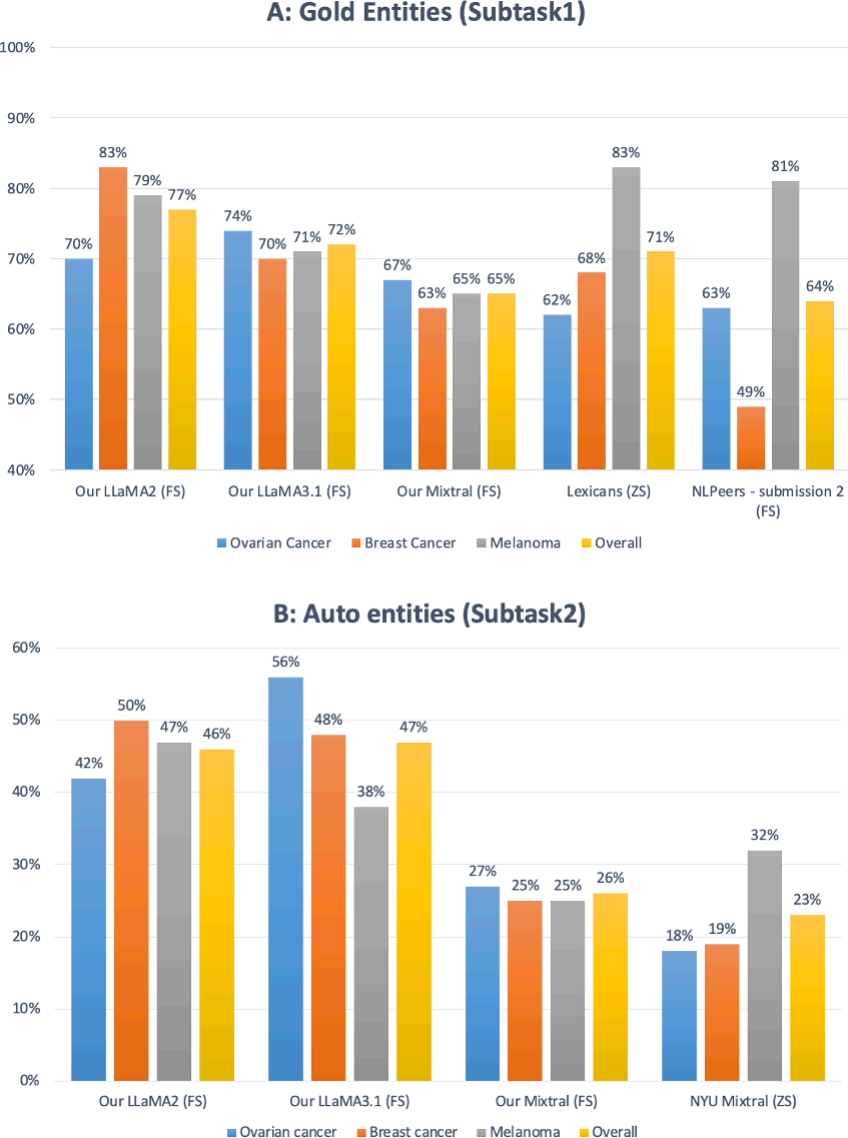
Comparison to LLM prompting systems in the shared task [13,17]. Scores are relaxed-to-month macro *F*_1_-scores on test set. “Our LLaMA2” and “Our LLaMA3.1” are LLaMA2-70B and LLaMA3.1-70B, respectively. “Our Mixtral” is the Mixtral-8 × 7B-Instruct-v1 model. FS and ZS refer to few-shot and zero-shot settings.

In Figure 4 we compare our LLM-based approaches with the shared task systems that prompted LLMs. With gold mentions as input (Subtask1), our system based on prompting LLaMA2 achieved the highest overall score compared to the shared task systems. When using Mixtral as the starting point, our system and the NLPeers [18] system achieved similar overall scores (65% vs 64%), which are significantly lower than the overall score of LLaMA2 and LLaMA3.1, suggesting that LLaMA family models are more suitable for this subtask than Mixtral. Only 1 team from the shared task explored end-to-end timeline construction using an LLM. In Figure 4-Part B, Subtask2 we can see that the overall performance of the two Mixtral-based systems is similar. Again, we see a performance discrepancy between LLaMA and Mixtral. Jiang et al [24] show that Mixtral performed better than or comparable to LLaMA2 across multiple benchmarks. Our results suggest that the decision of choosing the right LLM should be made empirically. Note that the two LLaMA models we used have the same number of parameters, 70B. Compared to LLaMA2, LLaMA3.1 improved the results on the ovarian dataset, but fell short on the breast and melanoma datasets. Across 64 evaluation settings (4 cancer types, 4 metrics, 2 subtasks, both development and test sets), LLaMA3.1 achieved higher or same *F*_1_-scores as LLaMA2 in 39 cases (61%; see Table S4 in Multimedia Appendix 1). Overall, we observe similar trends across strict, relaxed-to-day, relaxed-to-year evaluation settings as relaxed-to-month setting.

We performed error analysis on the relaxed-to-month output for each cancer type cohort. An incorrect prediction within a predicted patient timeline against a gold patient timeline is either a false positive, that is, a predicted triplet that is not present in the gold timeline, or a false negative, that is, a triplet in the gold timeline, which is not in the predicted timeline. There is also the possibility of an apparent false positive or false negative being actually correct due to an annotation error, for which we also review. We analyze which of the components in the system pipeline or the annotation process is the root cause of an error in the predicted or gold timelines. For the predicted timeline, this can consist of any combination of one of the extraction components for SACT EVENT mentions (SACT Detection Error) and temporal expression mention (TIMEX3 Detection Error), the TLINK classifier (TLINK Error), and summarization error (Total incorrect summarized predictions). For the gold timeline, this can only consist of an annotation error.

We present the breakdown per error type from the test set in Table S6 in Multimedia Appendix 1. We randomly sampled each type of false positive errors to collect a sample size using a 95% CI, a margin of error of 5%, and a population proportion of 50%. We analyzed the instance-level false positives since each was associated with a specific TLINK classification instance. The incorrect unsummarized predictions are inputs to the summarization algorithm which result in the incorrect summarized predictions. We found that most of the errors came from incorrect TLINK classification, followed by annotation errors, and finally detection of SACT EVENT and TIMEX3 mentions. We identified the annotation errors for the most part as resulting from likely missed screening of some notes by the expert annotators, as this is a highly cognitively demanding task for a human to perform (see Table 3 for examples). The false negatives tended to be the result of formatting issues, complex reasoning, and some level of hedging around the event. We found that in many notes, there are subsections that start with dates, which are used as the headings for these subsections (see examples in “False negative: formatting” in Table 3); then all events described in that subsection are related to these dates. This is especially challenging as the subsections could consist of multiple sentences.

**Table 3.** Types of errors and examples. Note that the dates have been intentionally altered for the purpose of this paper.

Type of error	Text	Explanation
Annotation error	Anastrozole (Arimidex) 1 mg once a day by mouth [Order Comment : can take am]. Last dose : 10/18/2033.	No gold TLINK^a^ for “anastrozole (Arimidex)” and “10/18/2033”.
Annotation error	Dr Person17, later today, to discuss management from the standpoint of chemotherapy or hormonal.	No gold TLINK for “later today” and “chemotherapy”.
Annotation error	Chemo and radiation in 2055.	No gold link for “chemo” and “2055”.
False negative: formatting	July through December 2055: Completed his 12 cycles of FOLFOX. The first 8 cycles included oxaliplatin and the last 4 cycles were 5-FU/leucovorin.	No prediction TLINK for “December 2055” and “5-FU/leucovorin”. The dates are used as subsection headings with all events related to them.
False negative: complex reasoning	November 2055, CEA begins to increase. There is abnormal uptake on a PET scan near the rectosigmoid junction. Patient is then initiated on XELIRI/Avastin in February 2055. [more text*.*.]. May 2055 through August 2055, managed with observation alone off of all chemotherapeutic administration.	No prediction TLINK to indicate that XELIRI/Avistin was discontinued May 2055 through August 2055.
False negative: hedging	We had attempted to treat him with ipilimumab last week; however, when he got the bathroom in the office, he tripped over a wheel of one of the beds and had a fall.	Gold TLINK is (last week, CONTAINS, ipilimumab). No predicted TLINK due to the expressed uncertainty of whether the event happened.
False positive: complex reasoning	…cycles of Cytoxan, fludarabine, and Rituxan chemotherapy through July 2055.	Predicted TLINKs are correct. However, the treatment is associated with the patient’s leukemia, not the melanoma which was the targeted extraction.

a TLINK: pairwise temporal relations.

## Discussion

### Principal Findings

The implications of the automatic and faithful extraction of treatment timelines from patients’ EMRs affect the spectrum of patient-physician interactions, decision-making processes, and advances in cancer research. At the point of care, a clinician presented with the patient’s treatment timeline would be able to quickly gain insights into the complex disease and treatment process for that patient, especially helpful in oncology where patients come to specialized centers with hundreds of notes. For research, the automatic generation of timelines opens the door to creating large-scale cohorts to answer important research questions. One such question is related to the treatment regimens as key details in understanding the effects of genetic, epigenetic, and other factors on tumor behavior and responsiveness. As precision oncology progresses, insights into the fine interplay of treatment with tumor molecular characteristics and patient phenotypes become even more critical not only as a source of research data, but as a means of translating findings into patient-tailored therapies similar to those that have been applied to breast cancer and melanoma [38].

Although there is a lot of excitement around LLMs and prompt engineering, there is a major constraint that needs to be factored into engineering decisions—that of the length of the input text. This is especially pronounced for tasks where the entire patient EMR narrative needs to be considered, for example, treatment timeline extraction. When considering the input prompt for LLMs, we first considered sending 1 note at a time to LLMs, or concatenating all the sentences that contain SACT EVENT mentions in a note and sending them to LLMs. However, our experiments showed that extracting timelines from long sequences (even just one patient note) was too challenging for the LLMs we evaluated (although these were the SOTA open LLMs). For example, on the ovarian cancer development set, we saw a 10-point drop in relaxed-to-month scores when we sent multiple sentences from the same document to LLaMA2.

As the error analysis pointed out, the main source of the error is TLINK classification, that is the assignment of the correct temporal relation between an EVENT and TIMEX. The technology we experimented with is LM-based—finetuning smaller LMs and LLM prompting. A path of research to improve TLINK extraction lies in combining the outputs of various technologies into an ensemble with a voting mechanism, for example, majority vote or a classification layer. The ensemble could potentially include the output of LLM-based and non–LLM-based methods such as classic support vector machines [39]. Another potential solution might lie in exploring a 2-stage LLM finetuning strategy, which is a refined ensemble method [40]. The first stage decreases bias and variance iteratively, while in the second stage, a selected fixed-bias model is used to further reduce variance due to optimization in ensembling. Soft prompting [41] might be another viable path to explore, especially given the availability of labeled data.

Our experiments show that LLMs struggle with end-to-end timeline extraction from clinical narratives (see Figure 4B). In Table 4, an examination of label distribution across the development set highlights a strong tendency of the system to overproduce BEGINS-ON and ENDS-ON relations while underrepresenting CONTAINS-1. For example, in colorectal cancer, the system predicted 381 BEGINS-ON and 281 ENDS-ON events, vastly exceeding the gold counts of 82 and 73, respectively. A notable source of error in the system’s predictions stems from confusion in relation directionality, particularly with the CONTAINS-1 relation. By design, all triples are structured as <EVENT, TLINK, TIMEX3>, where CONTAINS-1 semantically indicates that the drug was administered on the date specified by the TIMEX3 (see the Tasks and Datasets subsection in the Methods section). However, the system frequently reversed this logic, producing incorrect <EVENT, CONTAINS, TIMEX3> triples. Such mispredictions not only result in spurious labels (captured under the CONTAINS category in the label distribution) but also reflect a deeper modeling issue: the model’s difficulty in internalizing fine-grained relational semantics. To mitigate this, future work could incorporate explicit prompt instruction or soft constraints to enforce the expected directionality of relations during inference in the spirit of constrained decoding [42]. In addition, postprocessing steps could validate predicted relations by checking for allowable type-direction combinations, correcting or filtering those that violate domain-specific rules.

**Table 4.** Label distribution across the gold timelines and large language model (LLM) predicted timelines (LLAMA2 70B model, end-to-end setting) on the development set.

Cancer type	Gold timelines, n			System timelines, n			
	CONTAINS-1	BEGINS-ON	ENDS-ON	CONTAINS	CONTAINS-1	BEGINS-ON	ENDS-ON
Breast cancer	16	11	12	1	2	49	21
Ovarian cancer	65	8	12	7	11	104	38
Melanoma	39	5	1	2	8	47	22
Colorectal cancer	97	82	73	87	0	381	281

The error analysis also revealed incorrect annotations in the gold labels. We identified 30 annotation errors in the sample of the shared task dataset (~3.5 million words). The number of annotation errors in the CRC dataset sample is higher, but this is also the largest dataset (12 million+ words). Thus, as a proportion, the estimated annotation error rates across the independent datasets are similar. Annotation error is a standard hazard of the annotation process, especially for a highly cognitively demanding task as the timeline extraction from the entire patient’s chart. One has to review every single document from the patient’s chart, which for oncology patients translates into hundreds, if not thousands, of notes. Human errors are bound to happen. This further underscores the importance of developing methods for automatic and faithful timeline extraction.

A curious result emerges on the melanoma dataset. As shown in Table 2, the performance on the melanoma dataset is lower than the performance on other types of cancer using task-specific finetuned model. We believe this is caused by the data scarcity in the melanoma dataset because (1) SACT is not the main treatment modality for most melanoma presentations; therefore, there are fewer instances of SACT in the melanoma data and (2) the melanoma test set is the smallest of the 4 datasets. As the evaluation script computed the average *F*_1_-scores across all patients, the overall performance on the melanoma test set fluctuated greatly with the score of individual patients.

In this work, we focus on cancer treatment timeline extraction. However, the methodology described in this work can be applied to treatment timelines extraction of other diseases. For instance, if gold standard datasets are available for an out-of-domain disease type, one can finetune a small LM for temporal relation extraction. If gold annotations are not available for a type of disease, prompting LLMs with a few domain-specific examples would be a viable solution.

### Limitations

In this work, we did not use powerful, but proprietary LLMs such as GPT-4 [26] or Gemini [43], as we do not have access to nonretaining versions of these models for large scale processing. Despite the fact that our dataset was deidentified per HIPAA requirements, we did not feel that it was ethically appropriate to submit patient-derived data to a retaining LLM. However, experimenting with open models presents a realistic scenario for the average academic center as experimenting with proprietary LLMs comes at a significant cost. The LLMs we selected in our study were those reported to have competitive performance to proprietary models [24,36]. During paper revision, the DeepSeek-R1 [44] open model was released which outperformed the proprietary models on several general benchmarks. We leave experimentation with it as a future study. We did not use prompting techniques such as chain-of-thought [45] because it is not clear how to directly convert a complex task such as timeline extraction from the entire EMR clinical narrative into a series of reasoning steps. We leave the exploration of using HIPAA-compliant versions of proprietary LLMs (access-dependent) and other prompting methods such as prompt-tuning [46-48] for future research. Another limitation is that the datasets represent 2 medical centers and thus may introduce institutional or regional biases. However, to the best of our knowledge, these datasets are the only ones on cancer treatment timelines available to the community. In addition, this study focuses on colorectal cancer, breast cancer, ovarian cancer, and melanoma. While these common cancer types are broadly representative, future work should extend the SACT timeline extraction task to other cancer types. We should note that such pan-cancer extensions necessitate significant resources for the creation of the gold annotations. We also acknowledge that some cancer journeys are complex, with lines of therapy containing SACT interspersed with other therapeutic modalities such as radiation; these complexities are out of scope for the current approach but should be a focus of future work. Finally, this work uses an established set of predefined temporal relations (CONTAINS, BEGINS-ON, ENDS-ON, OVERLAP, and BEFORE) and preexisting annotations. We acknowledge that modeling more complex and nuanced temporal scenarios might potentially provide additional insights; however, this is the core set the clinical temporal information extraction community has converged on with some minor nuances [1,2,14].

### Conclusions

In this paper, we explored approaches for patient-level timeline extraction through the task of SACT timeline extraction. We performed experiments on the 2024 ChemoTimelines shared task as well as on the THYME dataset, thus the data represented 4 types of cancer across two institutions. We finetuned an LM that was specifically trained to attend to EVENT and TIMEX3 mentions. In that, we achieved higher scores than all shared task participants in Subtask1. We also explored LLM-based systems via prompting. In both subtasks, our LLM-based systems outperformed the shared task participant systems that took the approach of prompting LLMs. Our results contribute to the body of work that shows that task-specific finetuning based on rich, disease-specific datasets outperforms prompting the current generalist LLMs. We believe our results and analysis on this task add to the knowledge of extracting treatment timelines in EMRs using NLP methods. Our code will be released publicly upon acceptance.

## Supplementary material

10.2196/67801 Multimedia Appendix 1 More details on data statistics, experimental settings, results, and error analysis.

## Abbreviations

BIO: beginning, inside, outside
CRC: colorectal cancer
DocTime: Document Creation Time
DocTimeRel: relation with the Document Creation Time
EMR: electronic medical record
HIPAA: Health Insurance Portability and Accountability Act
LLM: large language model
LM: language model
NLP: natural language processing
SACT: systemic anticancer therapy
SOTA: state-of-the-art
THYME: Temporal Histories of Your Medical Event
TIMEX3: time expressions
TLINK: pairwise temporal relation
