# Supplementary material for: Systemic Anticancer Therapy Timelines Extraction From Electronic Medical Records Text: Algorithm Development and Validation
Source: JMIR Bioinform Biotechnol. 2025 Sep 3;6:e67801. doi: 10.2196/67801 (PMC12408058; doi:10.2196/67801)
Supplement: Multimedia Appendix 1 [file bioinform-v6-e67801-s001.docx]

Appendix Table 1. Label distributions of the gold train and development pairwise annotations across all cancer types. Of note, in the final SACT timeline, we converted the pairwise CONTAINS to CONTAINS-1 so that all triples are structured as <EVENT, TLINK, TIMEX3>, where CONTAINS-1 semantically indicates that the drug was administered on the date specified by the temporal expression (TIMEX3).

|  | Train | | | | | Development | | | | |
| --- | --- | --- | --- | --- | --- | --- | --- | --- | --- | --- |
|  | CONTAINS | BEGINS-ON | ENDS-ON | OVERLAP | BEFORE | CONTAINS | BEGINS-ON | ENDS-ON | OVERLAP | BEFORE |
| Breast  Cancer | 298 | 131 | 26 | 0 | 0 | 57 | 27 | 29 | 0 | 0 |
| Ovarian  Cancer | 327 | 101 | 66 | 0 | 0 | 140 | 34 | 52 | 0 | 0 |
| Melanoma | 37 | 10 | 1 | 0 | 0 | 157 | 42 | 2 | 0 | 0 |
| Colorectal Cancer | 2,209 | 2,047 | 1,636 | 0 | 5 | 910 | 509 | 494 | 11 | 0 |

Appendix Table 2. Experimental settings.

|  | Finetuning EVENT Tagger | Finetuning Temporal Relation Extractor | Prompting LLMs |
| --- | --- | --- | --- |
| Model | microsoft/BiomedNLP-BiomedBERT-base-uncased-abstract | https://physionet.org/content/entity-bert/1.0.1/ | meta-llama/Llama-2-70b-chat-hf  meta-llama/Llama-3.1-70B-Instruct  Mixtral-8x7B-Instruct-v1 |
| Learning Rate | 2e-5 | 4e-5 | / |
| Batch size | 32 | 32 | / |
| Number of epochs | 3 | 3 | / |
| Weight Decay | 0.01 | 0.01 | / |
| Computational Resource | NVIDIA Titan RTX GPU | NVIDIA Titan RTX GPU | NVIDIA A100 GPU |

Appendix Table 3. LLM prompting template for Subtask1, Step 1. The <EVENT> and <TIME EXPRESSION> tags are replaced by real entities in the experiments.

| Instruction: | You are a helpful assistant for oncologists. You will read a sentence with two entities highlighted: one is a chemotherapy treatment, the other one is a time expression. You are asked to classify the temporal relations between them. Please only generate outputs in the requested format. Do not include any other text or reasoning. Please do not use any labels outside [BEGINS-ON, ENDS-ON, CONTAINS-1]. |
| --- | --- |
| Query: | What is the temporal relation between <EVENT> and <TIME EXPRESSION>? |

Appendix Table 4: Evaluation results of our systems. Scores are macro F1 generated by the publicly available 2024 ChemoTimelines shared task evaluation script. Best results are in **bold**. Systems using small finetuned models are highlighted in gray.

|  | | Strict | | | Relaxed-to-Day | | | Relaxed-to-Month | | | Relaxed-to-Year | |  |  |
| --- | --- | --- | --- | --- | --- | --- | --- | --- | --- | --- | --- | --- | --- | --- |
| Cancer Type | Models | Dev | Test | Dev | | Test | Dev | | Test | Dev | | Test | |  |
| PART A: Subtask 1^a^ | | | | | | | | | | | | |  |  |
| Ovarian Cancer | EntityBERT | **88%** | 81% | **96%** | | **99%** | **93%** | | **95%** | **94%** | | **98%** | |  |
|  | EntityBERT (3 Cr)^b^ | 86% | **88%** | 94% | | 98% | **93%** | | 94% | **94%** | | **98%** | |  |
|  | LLaMA2 | 24% | 34% | 61% | | 63% | 70% | | 70% | 73% | | 76% | |  |
|  | LLaMA3.1 | 65% | 65% | 71% | | 72% | 75% | | 74% | 74% | | 88% | |  |
|  | Mixtral | 36% | 31% | 52% | | 55% | 60% | | 67% | 71% | | 70% | |  |
| Breast Cancer | EntityBERT | **94%** | 83% | **95%** | | **97%** | **97%** | | 97% | 97% | | **98%** | |  |
|  | EntityBERT (3 Cr) | 93% | **85%** | **95%** | | **97%** | **97%** | | **98%** | **98%** | | **98%** | |  |
|  | LLaMA2 | 61% | 42% | 74% | | 58% | 81% | | 83% | 82% | | 88% | |  |
|  | LLaMA3.1 | 69% | 54% | 73% | | 62% | 79% | | 70% | 84% | | 74% | |  |
|  | Mixtral | 56% | 37% | 59% | | 47% | 66% | | 63% | 75% | | 72% | |  |
| Melanoma | EntityBERT | **76%** | **76%** | **78%** | | **91%** | **86%** | | **91%** | **100%** | | **94%** | |  |
|  | EntityBERT (3 Cr) | 74% | 68% | **78%** | | 89% | **86%** | | 88% | **100%** | | 91% | |  |
|  | LLaMA2 | 65% | 45% | 77% | | 71% | 80% | | 79% | 98% | | 82% | |  |
|  | LLaMA3.1 | 51% | 56% | 62% | | 65% | 67% | | 71% | 85% | | 76% | |  |
|  | Mixtral | 50% | 53% | 61% | | 58% | 65% | | 65% | 84% | | 76% | |  |
| Colorectal Cancer (CRC) | EntityBERT | **84%** | **72%** | **90%** | | **79%** | **90%** | | **83%** | **88%** | | **89%** | |  |
|  | LLaMA2 | 45% | 55% | 56% | | 67% | 66% | | 77% | 77% | | 84% | |  |
|  | LLaMA3.1 | 52% | 55% | 58% | | 62% | 66% | | 68% | 75% | | 80% | |  |
|  | Mixtral | 39% | 48% | 49% | | 56% | 58% | | 66% | 67% | | 75% | |  |
| PART B: Subtask 2^c^ | | | | | | | | | | | | |  |  |
| Ovarian Cancer | EntityBERT | 48% | 56% | 53% | | 65% | 64% | | 61% | 76% | | 73% | |  |
|  | EntityBERT (3 Cr) | **51%** | **62%** | **58%** | | **68%** | **67%** | | **69%** | **77%** | | **86%** | |  |
|  | LLaMA2 | 2% | 28% | 19% | | 41% | 29% | | 42% | 41% | | 50% | |  |
|  | LLaMA3.1 | 15% | 30% | 24% | | 53% | 31% | | 56% | 45% | | 69% | |  |
|  | Mixtral | 1% | 19% | 2% | | 25% | 7% | | 27% | 18% | | 40% | |  |
| Breast Cancer | EntityBERT | 75% | 53% | **80%** | | 62% | **88%** | | 63% | **89%** | | 68% | |  |
|  | EntityBERT (3 Cr) | **76%** | **55%** | **80%** | | **64%** | 87% | | **66%** | **89%** | | **71%** | |  |
|  | LLaMA2 | 44% | 20% | 53% | | 32% | 61% | | 50% | 64% | | 53% | |  |
|  | LLaMA3.1 | 53% | 31% | 61% | | 42% | 66% | | 48% | 70% | | 52% | |  |
|  | Mixtral | 31% | 14% | 33% | | 20% | 37% | | 25% | 45% | | 31% | |  |
| Melanoma | EntityBERT | 38% | **31%** | 41% | | 34% | 43% | | 39% | 61% | | 45% | |  |
|  | EntityBERT (3 Cr) | **45%** | **31%** | 46% | | 35% | 46% | | 40% | **72%** | | 45% | |  |
|  | LLaMA2 | 18% | 25% | **51%** | | **43%** | **47%** | | **47%** | 46% | | **51%** | |  |
|  | LLaMA3.1 | 2% | 25% | 16% | | 33% | 26% | | 38% | 42% | | 46% | |  |
|  | Mixtral | 0% | 20% | 1% | | 23% | 4% | | 25% | 11% | | 28% | |  |
| Colorectal Cancer (CRC) | EntityBERT | **50%** | **40%** | **54%** | | **50%** | **58%** | | **56%** | **62%** | | **66%** | |  |
|  | LLaMA2 | 29% | 16% | 36% | | 27% | 40% | | 32% | 42% | | 40% | |  |
|  | LLaMA3.1 | 35% | 27% | 40% | | 35% | 45% | | 38% | 47% | | 48% | |  |
|  | Mixtral | 13% | 7% | 18% | | 13% | 19% | | 15% | 19% | | 17% | |  |

^a^Subtask 1: input is gold entities.

^b^EntityBERT (3 Cr): EntityBERT model trained only on the shared task data.

^c^Subtask 2: entities are automatically generated by our system.

Appendix Table 5. Event extraction results.

|  | Precision | Recall | F1 |
| --- | --- | --- | --- |
| Breast Cancer | 84.66 | 91.39 | 87.90 |
| Ovarian Cancer | 82.97 | 93.14 | 87.76 |
| Melanoma | 70.36 | 82.17 | 75.81 |
| Colorectal Cancer | 79.51 | 92.72 | 85.61 |

Appendix Table 6: Summarization of error types.

|  | Melanoma | Ovarian Cancer | Breast Cancer | CRC^a^ |
| --- | --- | --- | --- | --- |
| Annotation Error | 4 | 9 | 17 | 109 |
| SACT Detection Error | 0 | 0 | 1 | 3 |
| TIMEX3 Detection Error | 0 | 0 | 2 | 0 |
| TLINK Error | 20 | 36 | 117 | 305 |
| ‍‍Total incorrect unsummarized predictions | 24 | 45 | 137 | 518 |
| ‍‍Total incorrect summarized predictions^b^ | 20 | 25 | 86 | 176 |

^a^CRC: colorectal cancer.

^b^The incorrect unsummarized predictions are inputs to the summarization algorithm which result in the incorrect summarized predictions.
